# Exploring patient perspective: using narrative DIPEx interviews and the ICF model for interprofessional learning

**DOI:** 10.3389/fresc.2024.1424370

**Published:** 2024-10-01

**Authors:** Andrea Glässel, Ilona Hippold

**Affiliations:** ^1^ Department of Health Sciences, Institute of Public Health (IPH), Zurich University of Applied Sciences (ZHAW), Winterthur, Switzerland; ^2^ Unit DIPEx, Institute of Biomedical Ethics and History of Medicine (IBME), University of Zurich, Zurich, Switzerland

**Keywords:** patient-centeredness, interprofessional collaboration, interprofessional education, ICF, DIPEx, narration, patient perspective, health professionals

## Abstract

**Introduction:**

The International Classification of Functioning, Disability, and Health (ICF) has been widely adopted in academic health profession education and is part of bachelor curricula since its introduction by the WHO in 2001. In this context, interprofessional exchange among health professionals from a biopsychosocial perspective has become increasingly important and is now a key part of bachelor's program curricula to learn with, about, and from each other regarding students’ curiosity about interprofessional collaboration (IPC). This pilot study describes initial teaching experiences within an interprofessional elective module for health professions focused on patient-centeredness. It uses the ICF model to exemplify interprofessional exchange based on real patient experiences from the “DIPEx” database, which stands for “Database of Individual Patients’ Experiences.”

**Methods:**

Bachelor students from four healthcare professions learned in small interprofessional groups and selected case-related content from excerpts of real patient narratives from qualitative interviews in the DIPEx database. In a peer-to-peer process, students structured, analyzed, and reflected on selected patient experiences and presented their findings using the ICF model.

**Outcome:**

Develop a shared understanding of the case from a biopsychosocial perspective using the ICF model to communicate and reflect on patient-centeredness in interprofessional groups for a common care strategy rooted in patient-centeredness.

**Conclusion:**

This study illustrates how the shared analysis of a patient's experience of illness can lead to different perspectives on professional concepts for practice. The ICF model serves as a guiding structure and analysis tool. The core of the IPC, patient-centeredness, becomes the focus of the collaborative actions of the health professions.

## 1 Introduction

Since its adoption by the WHO in 2001, the International Classification of Functioning, Disability, and Health (ICF) model (1) has been widely used in the academic training of allied health professionals in bachelor curricula, such as nurses, occupational therapists, physiotherapists, speech therapists, and others, through various tools, training, and courses worldwide (2, 3).

Leonardi et al. provided a comprehensive review of ICF utilization over the past 20 years (2). The ICF model serves as an international standard tool for describing and measuring health status and disability. The article reflects on various aspects of the ICF model, including its application in the educational sector, which varies across countries. In Italy and Finland, individualized educational programs for students with disabilities have been adapted and implemented based on the ICF version for children and adolescents (ICF-CY). In Italy, the ICF is also used in the training of primary and secondary school teachers. In South Korea, a web-based tool was developed to assess the functional status of students with disabilities and to provide educational support. In Australia, the ICF has been proposed to develop resource allocation methods in the school system. Meanwhile, the ICF model is being integrated into various educational programs at universities, including programs, training workshops, and special conferences. ICF training has been developed and implemented in many countries.

The ICF is widely utilized in education, especially for adapting individual educational programs and developing assessment tools for students with disabilities. In several countries, the ICF is firmly integrated into educational programs. Despite its benefits, implementation challenges include a lack of commitment to ICF utilization and low awareness of its advantages at all levels. An obvious need exists for continuing professional development in ICF utilization. Online training, such as the ICF eLearning tool, enables training to occur anywhere and at any time. Notably, WHO-FIC Collaborating Centers support the development and dissemination of ICF training materials to ensure consistency in training.

The review by Leonardi et al. shows that the ICF is being used successfully in education and training in several countries (2). However, room for improvement remains in training and integrating the ICF into existing systems to fully realize its benefits. Ongoing support and training by the WHO-FIC Collaborating Centers markedly contribute to the dissemination and effective use of the ICF.

To more effectively and comprehensively comprehend complex health situations from a person-centered perspective, the ICF has become an increasingly crucial theoretical framework in bachelor’s degree programs and curricula based on the biopsychosocial model (3).

The focus is on practice-based interprofessional exchange rooted in patient-centeredness and a comprehensive perspective and common understanding (4). A current and comprehensive overview of ICF-based educational tools, as presented by the European INPRO project, underscores the importance of patient- or person-centeredness and the integration of educational experiences into daily practice (5).

To meet the complex needs of people in care, care must be tailored to individual needs, with person-centered care being essential for high-quality long-term care (6, 7). Person-centered care focuses on a therapeutic relationship rooted in respect, self-determination, and mutual understanding (8, 9). It positions patients as active participants in their care, shifting their role from passive to active in decision-making (9). Person-centered care also serves as the foundation for interprofessional collaboration (IPC) (10).

IPC is introduced early in bachelor programs to ensure high-quality patient care, which requires overcoming language barriers and integrating patient experiences, values, preferences, and goals (11). The WHO's 2011 Patient Safety Curriculum underscores the importance of patient-centeredness in health education (12). The Canadian Interprofessional Health Collaborative framework supports this by combining patient-centeredness with IPC (13). Incorporating real-world patient experiences into care is increasingly vital, as emphasized by the WHO's 2018 recommendations (14).

The WHO global patient safety action plan, presented at the 74th Assembly, highlights the need to involve patients in healthcare development and learn from their experiences to enhance safety (15) based on five strategies. Two of the five strategies are mentioned here in the context of education:

(a) Involving patients, families, and civil society organizations in the joint development of policies, plans, strategies, programs, and guidelines to enhance healthcare safety; and
(b) Learning from the experiences of patients and their families who have been exposed to unsafe healthcare to gain insight into the mechanisms of harm and to facilitate the development of more effective solutions.

Alford et al. (16) advocated for a person-centered approach that includes the biopsychosocial model for a better understanding of individual health experiences (17). This approach emphasizes patient narratives in healthcare decisions, reducing professional dominance (18). George Engel's biopsychosocial model from the 1970s also reflects this focus on personal narratives (19), including a person's autonomy.

Patient experiences, now routinely collected through questionnaires as standardized measurements (20) and narrative forms online, contribute to person-centered care. The DIPEx database, an online resource, allows patients to share their experiences, enriching the healthcare debate (21). The DIPEx captures the emotional, social, and psychological dimensions of illness, aligning with the ICF model (1). These narratives improve communication between patients and healthcare professionals, aiding decision making (22, 23). DIPEx serves as a quality-assured resource for health literacy and is part of an international network (www.dipexinternational.org) that spans 13 countries and offers over 100 topics on health and disease worldwide (24–29).

DIPEx was initiated by Andrew Herxheimer at the University of Oxford in 2000 and developed with Ann McPherson (21, 30). Their approach is characterized by an established qualitative research methodology, which is summarized in a handbook by the Health Experience Research Group (HERG) (24). The project's distinguishing feature is its systematic approach to collecting, analyzing, and presenting subjective real-world experiences of health and illness. This approach is based on scientifically defined, manualized, and peer-reviewed methods within the research network (24). This approach differentiates DIPEx from the various forms of patient narratives found on social media platforms such as forums, blogs, or commercial sites (31). The users of the national DIPEx websites as www.healthtalk.org, or www.DIPEx.ch, can be broadly categorized into five groups: patients and their families, health professionals, representatives of support groups, students of health and other professions, and others. National research groups have access to systematic insights into content and users via their statistics. The publication of authentic patient experiences is a freely accessible resource that provides crucial support for patients, relatives, clinicians in training, and educators in the health professions, as well as for research in various disciplines.

This case study aims to describe the initial interprofessional teaching experience based on an elective module for health professions at the bachelor's level, focusing on person-centeredness. It uses the ICF model to reflect on interprofessional exchange, drawing from real patient experiences in the DIPEx database.

## 2 Methods

This case study presents the development of an interprofessional online elective module entitled “Experiencing the Patient's Perspective Through Narrative Interviews,” which is worth 1 ECTS (European Credit Transfer System) for a group size of approximately 20–30 bachelor's students in their fourth semester (2023) from five health profession programs: physiotherapy, occupational therapy, midwifery, health promotion, and nursing. The students were permitted to select the module from a list of 30 different options and indicate a preference for one of the three available choices.

### 2.1 Students and faculty setting

The educational setting is interprofessional, with students learning in small groups (quartets) online. The content selected was case-related and drawn from excerpts of real patient narratives from qualitative interviews in the Swiss DIPEx database. Insights into subjective experiences of life and illness through narratives of patients and relatives can trigger reflection processes in students of health professions and support them in developing their professional self-image (32–34). Concerning the language of the ICF, this means transferring the subjective experiences of life and illness into an understanding of functioning and disability.

The teachers had backgrounds in health professions, including nursing, occupational therapy, and physiotherapy. They also had backgrounds in public health and applied ethics and were involved in interprofessional team teaching and facilitation. The team had experience in the conception of online teaching, joint interprofessional teaching, and acting as role models. They had many years of experience in training ICF content and interprofessional teaching. The author is a member of the Swiss DIPEx research team.

Students were required to complete a two-step group performance assessment process for this elective to be recognized as part of their education. The first stage is a plenary presentation by the small interprofessional groups at the end of the module. The second stage is a written reflection report as a group performance. The results will be reported transparently using a feedback Excel spreadsheet.

#### 2.1.1 Teaching methods and didactic approach

The teaching methods and didactic approach employed in this module encompass various learning formats. This encompasses blended learning for theoretical content, which employs e-sequences comprising voice-over video presentations, excerpts from book chapters, or scientific articles, among other formats. The latter is designed to facilitate the acquisition of basic knowledge, such as the ICF learning tool (www.ICF-learning-tool.org) (36). Three e-sequences on three main topics were provided for blended learning.

This course introduces the theoretical concepts of IPC, supported by a preselected collection of readings with practical examples. It also introduces the DIPEx database and its aims, teaching the basics of subjective theories of health and illness in the form of eLearning, followed by interactive webinars to discuss and deepen learning. Finally, it includes a learning diary to record the peer-to-peer learning experiences of learning from and with each other. The module did not prioritize the acquisition of research skills through the application of methods. Rather, methods were employed as a means to structure and condense the patients’ statements in terms of content.

The learning concept is based on interprofessional exchange and collaboration in small groups, beginning with the patient's perspective and utilizing the DIPEx data material. Emphasis is placed on shared reflection and the inclusion of the learning diary. The learning sessions in the small groups are supported by self-directed learning alternating with facilitated webinars.

The inclusion of live patients from DIPEx Switzerland allows students to directly engage with patient visitors and gain insight into their experiences of healthcare. This module is based on real-life experiences from the Swiss DIPEx database and provides insight into (a) the illness experience of patients with complex illnesses, functioning, and disability (e.g., chronic pain, blood cancer, pregnancy, dementia, and other topics) and (b) a qualitative analysis of themes related to subjective concepts of health and illness. Students can also work with DIPEx websites from other nations, such as www.healthtalk.uk or www.krankheitserfahrungen.de.

In the course, students work with the ICF model in its original form, entering information from component narratives in a straightforward manner. After an introduction to the ICF and its application (3, 36, 37), students are tasked with structuring, analyzing, reflecting on, and discussing care goals and strategies from both professional and interprofessional perspectives. To this end, they work in small interprofessional groups to process case material and place it in a professional context through dialogue and peer-to-peer feedback. Group reflection was supported by guided reflection in small groups with a teacher, aimed at bringing different viewpoints to the surface for discussion.

## 3 Results

A cohort of 24 students, aged between 22 and 30 years, comprising more women than men [23:1], was assembled from five academic programs: seven occupational therapists, five midwives, four nurses, three physiotherapists, and five health promotion students. The module commenced in March 2023 and was concluded in May of the same year.

### 3.1 Student sample and learning objectives

After eight weeks of interprofessional learning in 12 tandems and 6 small groups for peer-to-peer reflections and discussions, all 24 students passed the two-step assessment of the module successfully. This was achieved by presenting the results of the group process and discussing their experiences as well as submitting the reflection report. To address the learning process, all groups received a feedback Excel spreadsheet that outlined the learning aims and criteria for the oral presentation and written reflection reports.

#### 3.1.1 Step 1: learning outcomes assessment—oral presentation

The first step in the process of learning outcome assessment was an oral presentation. To gain an understanding of the collective process and outcomes of the module, students were required to document the main learning outcomes from 1 to 4, summarizing statements per small group on an online learning page. This was done to facilitate discussion of the overarching challenges, benefits, and learning outcomes based on guided questions.

One group elected to focus their efforts on the DIPEx topic of health experiences with epilepsy. They formulated the following statements:

Statement 1: There are numerous types of seizures.
Statement 2: The patient's perspective is of paramount importance in therapy.
Statement 3: Each patient must be considered individually; the intervention is not solely based on the diagnosis.
Statement 4: Life with epilepsy can be markedly different. In light of these considerations, there may be instances where limitations are either pronounced or minimal (Module- spring 2023, group 1).

A second group of students decided to work on the DIPEx-based experiences with palliative care. They formulated the following statements:

Statement 1: Acceptance is an important step in coping with terminal illness.
Statement 2: The ability to make one's own decisions about assistance gives people autonomy and a sense of independence.
Statement 3: The social environment plays an important role in well-being and can be very individual.
Statement 4: As a healthcare professional, one can make a significant impact on the life of a palliative patient by adapting to their individual needs during their care. (Module—Spring 2023 Group 2).

A third group of students opted to focus their efforts on the DIPEx-based experiences surrounding neonatal death. Their subsequent statements were as follows:

Statement 1: The necessity of a common language (interprofessional) to ensure the delivery of optimal care for patients.
Statement 2: The importance of viewing patients as individuals rather than merely medical cases (biopsychosocial).
Statement 3: The patient's perspective is a crucial foundation for reflection among health professionals. (Mirror of our work).
Statement 4: The patient perspective provides an opportunity to critically examine our actions. (Module—Spring 2023 Group 3).

After the presentation, each group presented a brief statement summarizing their learning impressions and feedback to the entire group and the facilitators.

#### 3.1.2 Step 2: learning outcome assessment based on the reflection report

All six small groups submitted their reflection reports, which were evaluated based on their content and presentation of the learning objectives (see Table 1) for IPC in small groups with a patient-centered focus.

**Table 1:** Learning objectives of the module: the students will be able to.

(1) Reflect on their professional actions and develop a first professional self-image based on existing case-related narratives (selection: chronic pain, multiple sclerosis, Parkinson's disease, COVID-19, dementia, prenatal diagnostics, and other topics), considering subjective theories of health and illness. (2) Apply basic knowledge of qualitative analysis of content in small interprofessional groups. (3) Identify health-related attitudes, question motives and patterns of action, and recall the fundamentals of subjective theories of health and illness. (4) Compare their theoretical knowledge with their own experiences and the knowledge of other professions and reflect on their values on a case-by-case basis. (5) Practice qualitative analysis based on six steps of thematic analysis (35) applied to existing transcripts, exploring themes and reflecting on their usefulness for professional practice. (6) Completing tasks on time and according to the assignment, both independently and through interprofessional dialogue, is also required.

The students described the main learning outcomes as follows:

- The ICF used as a model has so far been a theoretical tool without a clear statement on how to use it or how it can be used. However, after this combination with the DIPEx experiential examples, it becomes clearer how the benefits can be understood as a practical tool and easily applied in practice.
- Interprofessional discussions based on the patient's perspective can reveal the capacity for collaboration and mutual learning. These discussions facilitate the acquisition of knowledge from and about each other, thereby enabling the development of a shared understanding.
- The use of authentic patient narratives based on transcripts, audio-recording, and video as a foundation for discourse is both compelling and surprising in its portrayal of the multifaceted ways in which individuals conceptualize, anticipate, and fret about the implications of treatment and healthcare strategies.
- Students articulate new insights, challenges, and beneficial aspects of their skills and practical work with patients and families that they were previously unaware of.
- In the future, students will return to the DIPEx websites to prepare for their placements and to familiarize themselves with the expectations of patients and carers. Furthermore, reflection and debriefing from the trainers’ perspective on teaching and facilitation will be provided.

Overall, the primary learning objectives were effectively met by all groups. The learning environment was conducive to ICF utilization in future patient settings.

### 3.2 Evaluation process of the module

The elective module, “Experiencing the Patient's Perspective Through Narrative Interviews,” was evaluated by the standard process of the ZHAW Office for Interprofessional Learning. This evaluation was conducted using the university's system via the Moodle platform, which included both open-ended and closed-ended questions. The response rate for this course was 60%, which represents two-thirds of the opinions of the students who attended the module. This response rate is within the acceptable range for module evaluations conducted by standard procedures.

The evaluation results indicated that the forms of teaching and learning, content structure, level of demands, and workload were rated as satisfactory, suggesting no need for immediate action in this regard. However, the clarity of the learning objectives and module organization were assessed somewhat more negatively. This could be interpreted as the perceived lack of clarity and uncertainty regarding the performance record, as evidenced by the open answers. Therefore, determining the extent to which improvements in this area would lead to a more transparent and coherent learning experience for students, as well as a more structured and effective module, is necessary. The extent to which other factors may have influenced this outcome and the necessity for further action must be determined by the module coordinator.

## 4 Discussion and conclusion

The discussion of this case study elucidates the utilization of the ICF model as a patient-centered approach in an interprofessional learning experience within a bachelor's level elective module for health professions. The emphasis is on using the ICF model as an exemplar to facilitate reflection on interprofessional exchange through authentic patient experiences from the DIPEx database.

The initial results of a randomized controlled trial study of patient stories from the DIPEx database indicated that students benefited from real patient narratives and performed more competently in terms of their communication skills than students in a comparison group who were taught the content by experts (38). Additionally, evidence from interprofessional learning settings (physiotherapy and social work) supports these initial indications (39). Narratives represent a potential approach to experiences by facilitating a patient-centered perspective regarding the inner experience of illness, experiences with the care system, treatment outcomes, and coping with illness (40).

The following learning example from a pilot study also describes the integration of the patient's perspective into everyday clinical practice. To work in a targeted and effective way, (future) clinicians should learn to understand the patient's perspective on their illness. The subjective experience of illness can be described in terms of the personal accounts presented in illness narratives (41). Such illness narratives can be found on the DIPEx website. A pilot study describes a teaching unit developed using videos of weight loss in patients with type 2 diabetes. This pilot study aimed to teach medical students to recognize different subjective views and reactions to patients’ previous lifestyle choices. The study explored how students and faculty learned to better understand patients’ perspectives based on disease reports, although external conditions are the biggest challenge for patients who need to change their lifestyles (42).

Regarding the enhancement of patient-centeredness, Charon (2007) (43) and Shao-Yin et al. (2020) (44) demonstrated that engaging in active listening is a pivotal element influencing the practitioner–patient relationship. This is associated with various positive outcomes, including appreciation, respect, and empathy. For instance, Care Ethics by Joan Tronto offers a valuable framework for healthcare professionals to reflect on ethical issues and questions in practice related to patients’ needs (45). Video, audio, or text contributions from systematically collected online patient narratives can support educators in strengthening this ability in students, which can be considered a subcomponent of narrative competence. Additionally, experience reports are now being used to design case vignettes and to prepare simulated persons in medical and health professions training for staging to achieve a higher degree of credibility, persuasiveness, and identification with the role to be conveyed (46).

Since the spring semester of 2024, medical students at the University of Zurich (UZH) have been learning together with students from the health professions at the Zurich University of Applied Sciences (ZHAW) in a DIPEx-based module, dealing in an exemplary manner with experiential perspectives and practicing patient-centered active listening and interprofessional dialogue with each other.

### 4.1 Strengths and limitations

The case-based application of the ICF model using real patient experiences from the DIPEx database supported the students in applying and understanding the ICF components as an interaction model. The students found the sheet to be simple and clear, with direct reference to practice. They were able to reflect on and discuss professional and interprofessional issues based on the patient's perspective. One disadvantage they identified was that the DIPEx database did not contain complete case descriptions. They felt that this might result in the omission of essential information that would be necessary to complete a comprehensive picture of a patient. This issue was also identified in practice, as obtaining all the information about a person during a first or second treatment session is not possible. The necessity for a complete picture of the patient develops over time. In this context, the benefits of learning from each other were identified, as well as the opportunity to gain insight into each other's roles as health professionals through a patient-centered approach, facilitated by the ICF model.

The initial implementation of the learning offer was advantageous in that the number of students was less than 30. This allowed the interprofessional team of facilitators to respond in a highly specific manner to the learning experiences and questions of the students in the small groups. The students confirmed this with an open atmosphere and found an approach to the topic. In a second circle, more students will be offered the module, and different group dynamics will be expected.

The lessons learned will be incorporated into a second implementation, and the learning objectives will be more thoroughly explained to the students. The open-ended learning task of researching and selecting patient experiences from the extensive DIPEx database initially challenged and sometimes overwhelmed the students, especially since they had not yet interacted with patients as part of their studies. However, this experience offers the potential for both DIPEx and the students to prepare for future patient interactions, allowing sufficient time to address questions, behaviors, and reactions.

### 4.2 Lessons learned and conclusion

- Students and facilitators reflected on aspects of the lessons learned following this inaugural interprofessional learning experience based on the ICF. The incorporation of patient voices is increasingly recognized as a crucial factor in the continuous improvement of healthcare. The inclusion of patient experiences through narratives appears to be of growing importance, given the life-changing consequences for patients and their families.
- Not all students from the five programs were fully familiar with the ICF and its application. To establish a foundation for collaborative learning, the introduction can be improved to ensure that all members of the small groups start from a common point. To achieve this, a revision is recommended to enhance the depth of knowledge or to reflect the existing knowledge on different concepts more precisely within the group.
- Health profession students reported a benefit from a clear and simple form of the ICF model, which provided a practical means of describing even complex contexts and relations between the ICF components. They also appreciated the change in perspective within the small interprofessional groups.
- The interprofessional dialogue was encouraged and practiced in a low-threshold manner, and there was sufficient time to deepen different learning experiences, including theoretical concepts related to the student's respective professions, through the peer-to-peer approach. The learning diary has not yet been used extensively.
- Health profession students summarized that patients’ descriptions of their individual experiences in dealing with illness and health are an essential approach to understanding and supporting comprehensive care-relevant goals, motivations, attitudes, participation, or even decisions. For person-centered care to be effective, gaining insight into patients’ experiences is essential.
- One way to achieve this is to actively include patient voices in the teaching process. This approach, which can be considered a methodological-didactic strategy, encourages students to reflect on their perspectives and understanding and to consider the authenticity and realism of their actions. It also prepares students for practice by allowing them to engage in critical thinking and action.

This case study on collaborative learning illustrates how the joint analysis of a patient's experience of illness, functioning, and disability can lead to different perspectives on professional concepts for practice and collaboration. The ICF model serves as a guiding structure based on the ICF components and analysis aids for the students. The core of the IPC, person-centeredness, is trained and stimulated by learning tasks, including guided reflection on different levels. Person-centeredness becomes the focus of the collaborative actions of the health professions. Real patient experiences of functioning and illness are compelling due to their authenticity. Each learning objective could be further deepened, serving as a foundation for shared learning from and with patients for future practice. Based on these insights, an interprofessional module is proposed for the spring of 2025, focusing on the lessons learned from both the students’ and facilitators’ perspectives.

## Data Availability

The datasets presented in this article are not readily available because data come from education modules. Requests to access the datasets should be directed to andrea.glaessel@zhaw.ch.
